# Do we have an opioid crisis in Scandinavia? Time to act?

**DOI:** 10.1080/17453674.2018.1465285

**Published:** 2018-05-01

**Authors:** Marcus Heilig, Magnus Tägil

**Affiliations:** a Center for Social and Affective Neuroscience, Department of Clinical and Experimental Medicine, Linköping University, Linköping, Sweden;; b Department of Orthopedics, Clinical Sciences, Lund University and Skåne University Hospital, Lund, Sweden

In the United States, the prescription of opioids has quadrupled since 2000. This has been paralleled by a steady rise in the rate of opioid-related deaths, now reaching an estimated 65,000 American lives ended yearly by drug overdoses (Nature 2017). About 30,000 of these are due to legally prescribed drugs. Attempts to clamp down on prescriptions have done little to halt the trend, and instead in the past 2 years synthetic opioids, dominated by fentanyl and fentanyl derivatives, have accounted for an even steeper rise in overdose deaths. It is reasonable to ask whether the opioid crisis is a unique US problem, or whether we might face the same problem internationally, and in particular in Scandinavia, presently or in the years to come?

The question is in part addressed by a study by Bäckryd et al. (2017). The analysis indicates that in Sweden the prescription of opioids has remained at a constant level since the turn of the century. The total prescription of opioids, measured as morphine equivalents and defined as daily doses, remained constant between 2000 and 2015. Also, the number of patients who at some period during the year were taking opioids remained constant from 2006 to 2015, as did the amount of opioids prescribed per person. The authors therefore concluded that there is no opioid crisis in Sweden similar to what the US is experiencing. This does not invalidate reports that dependence on prescription opioids is a real clinical problem. That 12% of Swedes were receiving an opioid at some period in 2015 may not represent an increase, but is still a lot. Furthermore, the study indicates a shift toward short-acting opioids, such as oxycodone and fentanyl, which are known to have a higher addictive potential.

The orthopedic community should aim to minimize unnecessary opioid exposure, especially as opioids are relatively ineffective as analgesia for non-acute, non-cancer pain. In fact, in chronic pain, opioids are never the primary choice. In acute pain episodes, opioids have become increasingly popular, but even here evidence is scant (Brummett et al. 2017). In the summer of 2017, an enquiry was sent to the 6 university and 13 larger hospitals in Sweden that perform surgery on distal radius fractures. The study was an international initiative to evaluate prescription patterns after distal radius surgery, and the results were presented at a symposium on the opioid crisis held at the American Society for Surgery of the Hand in San Francisco in September 2017. All Swedish centers routinely prescribed the full dose of paracetamol. 10/15 prescribed additional short-acting oxycodone (n = 10), tramadol (n = 1), or paracetamol + codeine (n = 1), in packets of either 14 or 28. 3 centers gave out drugs only for 3 days. 10/15 centers prescribed long-acting oxycodone in packets of either 14 or 28. Repeat prescriptions were reported as rare. The Swedish prescription pattern was found to be identical to the enquiry made among American centers.

It thus appears that we do not have an ongoing full-scale opioid crisis in Sweden, at least not of the same catastrophic magnitude as the United States. However, we need to be aware of the risk that ill-indicated prescription of opioids, and in particular short-acting members of this group, may also lead patients into a lifelong opioid addiction in Sweden. A recent study from Denmark has found a considerable proportion of patients with continued or increased opioid consumption 1 year after hip or knee replacement (Jørgensen et al. 2018). It is plausible that we need to act now to stop a future crisis. Our postoperative prescription patterns match the US practice, which has obviously led to a national public-health emergency. It is naive to believe we are immune to a similar deterioration in Scandinavia.
